# Influence of electric current pulses on the solidification of Cu-Bi-Sn immiscible alloys

**DOI:** 10.1038/srep12680

**Published:** 2015-07-31

**Authors:** Jiang Hongxiang, He Jie, Zhao Jiuzhou

**Affiliations:** 1 Institute of Metal Research, Chinese Academy of Sciences, Shenyang 110016, China

## Abstract

Continuous solidification experiments were carried out with Cu-Bi-Sn alloys under the effects of Electric Current Pulses (ECPs). A model describing the microstructure evolution was developed. The formation of the microstructure in the continuously solidified alloys was calculated. The calculations demonstrated that ECPs mainly affect the solidification process through changing the energy barrier for the nucleation of the minority phase droplets (MPDs). When the matrix liquid has a lower electric conductivity compared to the MPD, the ECPs lead to a decrease in the energy barrier for the nucleation of the MPDs which then promote the formation of a finely dispersed microstructure. When the matrix liquid has a higher electric conductivity compared to the MPD, the ECPs cause an increase in the energy barrier for the nucleation and lead to the formation of a phase segregated microstructure.

Many alloys show a phase diagram characterized by the appearance of a miscibility gap in the liquid state. Some of them have great potentials to be used in industry, such as Cu-Pb, Al-Pb or Al-Bi, which could be potential materials for advanced bearings in automotive applications if the soft Pb or Bi phase is dispersed in a hard matrix like Al or Cu^1^. But when a homogeneous liquid is cooled into the miscibility gap, the components are no longer miscible and then two liquid phases develop. Generally, the liquid-liquid decomposition begins with the nucleation of the liquid minority phase in the form of droplets. These droplets grow and coarsen. They can also settle or float due to specific gravity differences between phases and migrate due to temperature gradients. The droplets motion causes the formation of a microstructure with great phase segregation. To overcome this drawback, plenty of efforts have been made to investigate the kinetics of the liquid-liquid decomposition^2^,^3^,^4^,^5^,^6^. It has been demonstrated that some solidification techniques have great potential in manufacturing immiscible alloys with finely dispersed microstructures^7^,^8^,^9^. Researches also indicated that external fields, i.e. static magnetic field^10^, centrifugal force field^11^, microgravity field^12^ and direct current^5^,^13^ may show great effects on solidification behavior. Recently, Zhu *et al*.^14^,^15^ carried out solidification experiments with Al-6.5 wt.% Bi immiscible alloys under the effects of Electric Current Pulses (ECPs). They reckoned that the ECPs caused the Bi-rich droplets to move towards the surface of the sample causing collision and coagulation between droplets and the formation of the segregated microstructure phase. In this paper, continuous solidification experiments are carried out with immiscible alloys under the effect of the ECPs. A model has been built to describe the microstructure evolution in a continuously solidified immiscible alloy. The solidification behavior of immiscible alloys under the effects of the ECPs as well as the affecting mechanisms of the ECPs on the microstructure evolution of the immiscible alloys, are discussed in detail.

## Results

**Figure 1:**
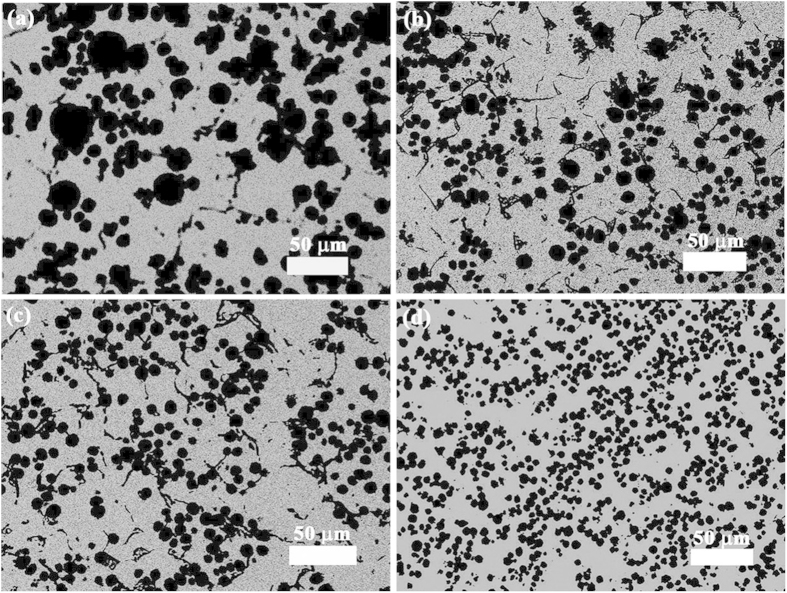
Effect of the ECPs on the microstructure of Bi-10%Cu-10%Sn alloy solidified at the rate of 10 mm/s. (**a**) without the ECP treatment, (**b**–**d**) with the ECP of the peak current density of 1 × 10^8^ A/m^2^ (**b**), 2 × 10^8^ A/m^2^ (**c**) and 3 × 10^8^ A/m^2^ (**d**), respectively. The frequency of the ECPs is 50 Hz. The duration of each electro-pulse is 6 *μs*.

**Figure 2:**
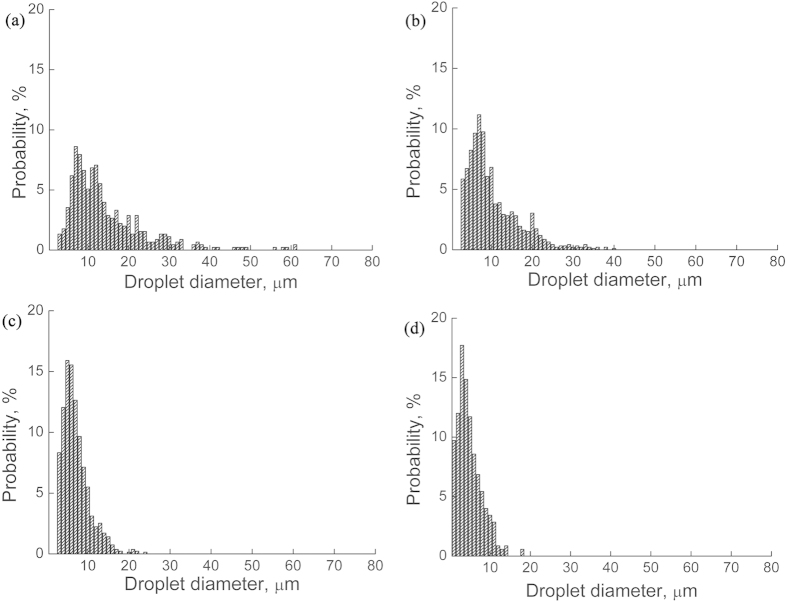
Size distributions of the minority phase particles in Bi-10%Cu-10%Sn alloy solidified at the rate of 10 mm/s. (**a**) without the ECP treatment, (**b**–**d**) with the ECP of the peak current density of 1 × 10^8^ A/m^2^ (**b**), 2 × 10^8^ A/m^2^ (**c**) and 3 × 10^8^ A/m^2^ (**d**), respectively. The frequency of the ECPs is 50 Hz. The duration of each electro-pulse is 6 *μs*.

Figure 1 shows the microstructures of the Bi-10%Cu-10%Sn (wt.%) samples solidified at the rate of 10 mm/s with ECPs of different current densities, and Fig. 2 shows the size distributions of the dispersed particles in the samples. The energy dispersive X-ray spectroscopy (EDS) analyses indicated that the white phase and the dark phase are the Bi-rich matrix phase and the (Cu,Sn)-rich minority phase, respectively. Figures 1 and 2 demonstrate that the number density of the dispersed particles increases, the average size and the width of the size distribution of the dispersed particles decrease with the increase in peak values of pulse current density. In other words, for the Bi-10%Cu-10%Sn alloy, the ECPs result in the formation of a sample with finely dispersed microstructure.

**Figure 3:**
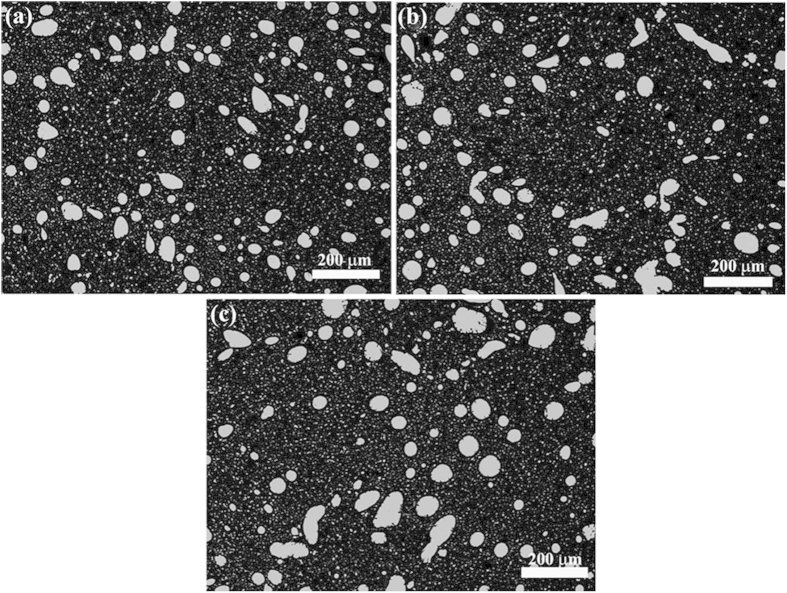
Effect of the ECPs on the microstructure of Cu-25%Bi-25%Sn alloy solidified at the rate of 10 mm/s. (**a**) without the ECP treatment, (**b**) and (**c**) with the ECP of the peak current density of 1 × 10^8^ A/m^2^ (**b**) and 3 × 10^8^ A/m^2^ (**c**), respectively. The frequency of the ECPs is 50 Hz. The duration of each electro-pulse is 6 *μs*.

**Figure 4:**
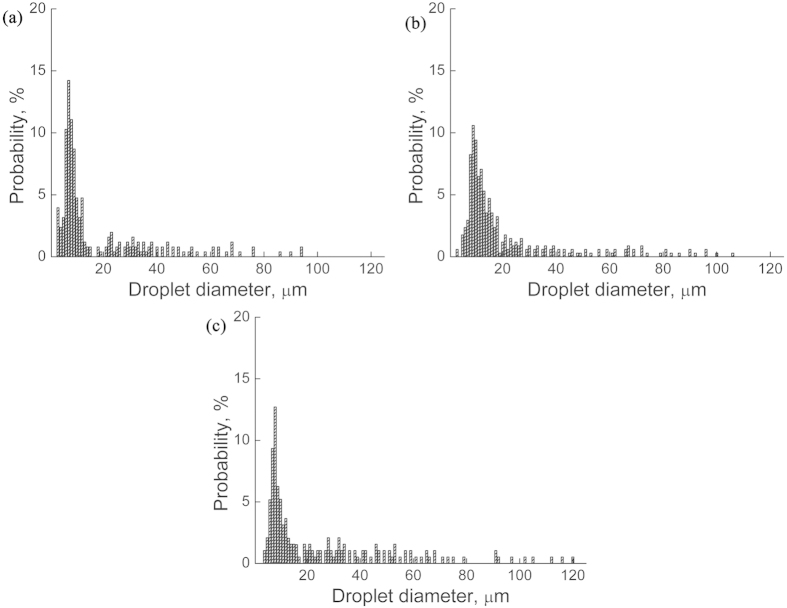
Size distributions of the minority phase particles in Cu-25%Bi-25%Sn alloy solidified at the rate of 10 mm/s. (**a**) without the ECP treatment, (**b**) and (**c**) with the ECP of the peak current density of 1 × 10^8^ A/m^2^ (**b**) and 3 × 10^8^ A/m^2^ (**c**), respectively. The frequency of the ECPs is 50 Hz. The duration of each electro-pulse is 6 *μs*.

Figure 3 shows the microstructures of the Cu-25%Bi-25%Sn (wt.%) samples solidified at the rate of 10 mm/s under the effect of the ECPs, and Fig. 4 shows the size distributions of the dispersed particles in the samples. It indicates that the number density of the dispersed particles decreases, the average size and the width of the size distribution of the dispersed particles increase with the increase in peak values of pulse current density. That is, for the Cu-25%Bi-25%Sn alloy, the ECPs prevent the formation of a sample with well dispersed microstructure.

## Discussion

The microstructure evolution during cooling in the miscibility gap in the liquid state is a result of the concurrent action of the nucleation, diffusional growth and motions of the MPDs. We describe the microstructure by defining a droplet radius distribution function *f*(*R*, *r, z, t*). *f*(*R*, *r, z, t*)*dR* gives the number of the MPDs per unit volume at position (*r*, *z*) and time *t* between droplet radius *R* and *R* + *dR*. It obeys the following equation during solidification:


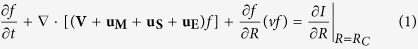


Where **u**_**E**_ is the migration velocity of the MPDs due to the electric current. **u**_**S**_ & **u**_**M**_ are the Stokes and Marangoni velocities of the MPDs. **V** is the moving velocity of the melt. *v* is the growth or shrinkage rate of the MPDs. *R*_*C*_ is the critical nucleation radius of the MPDs and *I* is the nucleation rate. It can be calculated by using the classical nucleation theory^16^. The free energy change due to the nucleation of the MPDs is given by^17^:


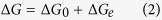


Where Δ*G*_0_ is the change of free energy in a current-free system. Δ*G*_*e*_ is the change of free energy due to the ECP. It is given by^18^:


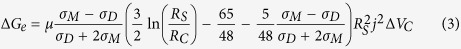


Where *μ* is the permeability of the melt. Δ*V*_*C*_ is the volume of a nucleus. *R*_*S*_ is the diameter of sample. *σ*_*D*_ & *σ*_*M*_ are the conductivities of the droplets and matrix, respectively. *j* is the electric current density. It can be calculated by 
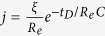
 in each pulse duration, where *ξ* is the discharging voltage. *R*_*e*_ is resistance. *C* is capacitance and *t*_*D*_ is the discharging time.

The ECP may distribute non-homogeneously over the cross section of the sample due to the skin effect. The skin depth on which the current density is mainly focused can be described by 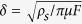
19, where *ρ*_*s*_ is the resistivity of the melt and *F* is the frequency of the ECP. For our experiment, the calculated skin depths are about 8.1 cm for the Bi-10%Cu-10%Sn alloy and 5.9 cm for the Cu-25%Bi-25%Sn alloy. They are both much larger than the radius of the sample (3 mm). We thus conclude that the ECP distributes homogenously over the cross section of the sample.

The temperature field of the sample is determined by the energy conservation relationship:


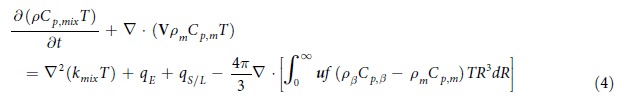


Where ***u ***= **V** + **u**_**M**_ + **u**_**S**_ + **u**_**E**_ represents the migration velocity of the MPDs. *ρ* is the density of the alloy. *ρ*_*m*_ & *ρ*_*β*_ are the densities of the matrix melt and the MPDs, respectively. *k*_*mix*_ & *k*_*p,mix*_ are the thermal conductivity and specific heat of the melt, respectively. *C*_*p,β*_ & *C*_*p,m*_ are the specific heat of the MPDs and the matrix liquid, respectively. *q*_*E*_ is the joule heat released per second. *q*_*S/L*_ is the latent heat released at the S/L interface per second and *T* is temperature.

The concentration field is determined by the solute conservation relationship:


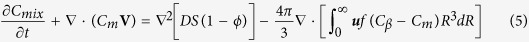


Where *C*_*mix*_ is the concentration of the melt. *C*_*m*_ is the mean field concentration of the matrix liquid. *S *= *C*_*m*_ − *C*_*me*_ is the super-saturation of the matrix liquid, *C*_*me*_ is the equilibrium concentration of the matrix liquid. *C*_*β*_ is the concentration of the liquid within the MPDs. *D* is the diffusion coefficient and *ϕ* is the volume fraction of the MPDs.

The flow field of the melt is determined by the continuity equation for an incompressible fluid and the Navier-Stokes equations:


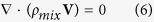



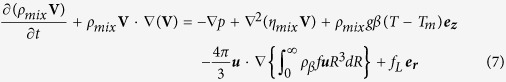


Where ***e***_***r***_ is the unit vector in the radial direction and ***e***_***z***_ is the unit vector in the axial direction. *p* is pressure. *β* is the thermal expansion coefficient. *η*_*mix*_ is the viscosity of the melt, *T*_*m*_ is the monotectic reaction temperature, 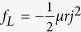 is the density of the Lorenz force due to the electric current and *g* is the gravitational acceleration.

The microstructure evolution in the continuously solidified Cu-Bi-Sn alloys was calculated by solving equation (1) together with equations (2) through (7), 3, 8, 9, 8, 9. The numerical method used in this analysis was the finite volume method^20^ and the calculation was done in 2D axisymmetric cylindrical coordinates. The radial axis and axial axis of the sample and the droplet radial axis were divided into a number of intervals, which constitute the finite volumes. Equation (1) was discretized as an implicit difference equation using the assumption that the droplet radial distribution function varies in a stepwise manner in the direction of the droplet radial axis (i mesh points) as well as in the directions of the radial axis (m mesh points) and axial axis (n mesh points) of the sample. The discretization of the continuous equation yields i × m × n ordinary differential equations with one equation for each of the i × m × n finite volumes. These equations were solved using the line-by-line algorithm, which is a combination of the algorithm of Gauss-Seidel and that of Thomas^20^. The main physical parameters used in the calculations, such as the diffusional coefficients, thermal conductivities and electric conductivities, etc. can be found in references^21^,^22^,^23^,^24^.

**Figure 5:**
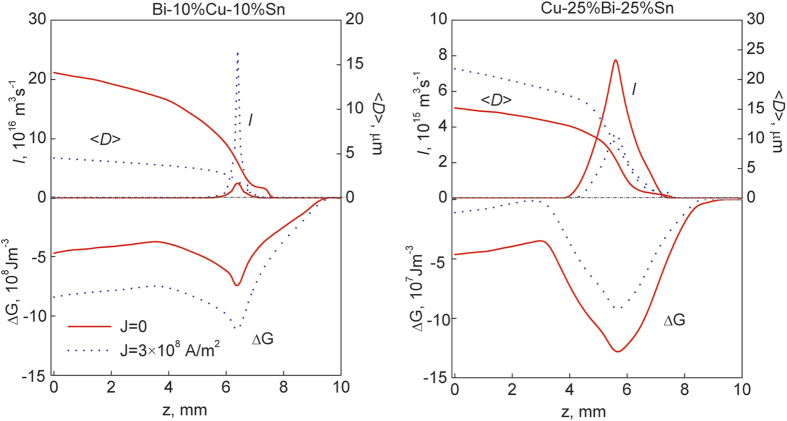
Nucleation rate (I) and average diameter (<D>) of the minority phase droplets, and driving force for the nucleation (ΔG) of the minority phase droplets when that alloys solidifies continuously at the rate of 10 mm/s under the effect of the ECPs of different current densities. The frequency of the ECPs is 50 Hz. The duration of each electro-pulse is 6 *μs*.

**Figure 6:**
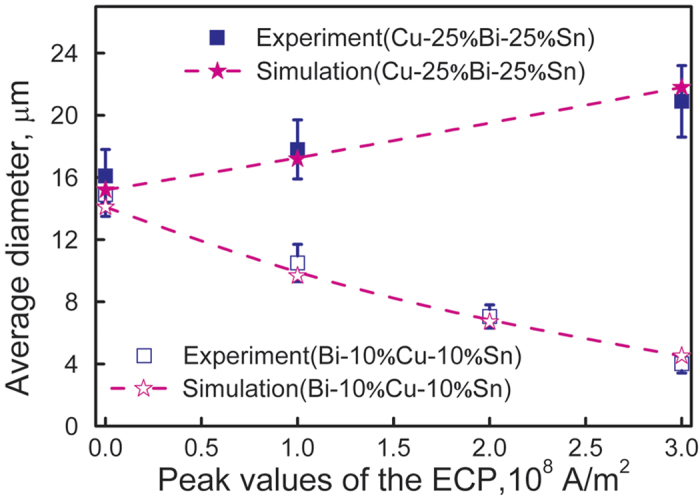
Average diameter of the minority phase particles in the samples solidified under the effect of the ECPs of different current densities.

The numerical results indicate that the joule heat show a negligible effect on the temperature profile in front of the solidification interface. Also, the effects of the Lorenz force on both the motion of the MPDs and the convective flow of the matrix liquid are negligible. The ECPs mainly affect the microstructure formation through changing the nucleation behaviors of the MPDs. For the Bi-10%Cu-10%Sn alloy, the (Cu,Sn)-rich phase droplets precipitate in the Bi-rich matrix liquid during the liquid-liquid decomposition and the matrix liquid has a lower electric conductivity compared to the MPDs. It is evident from the equation (3) that the energy barrier for the nucleation of the MPDs decreases with the application of an electric current (Δ*G*_*e*_ < 0). This decrease in energy barrier leads to an increase in the nucleation rate of the MPDs and ultimately a reduction in average diameter of the minority phase droplets/particles, as shown in Figs 5 and 6. For the Cu-25%Bi-25%Sn alloy, the Bi-rich minority phase droplets precipitate in the (Cu,Sn)-rich matrix liquid. The matrix liquid has higher electrical conductivity compared to the MPDs. An electric current leads to an increase in the energy barrier for the nucleation of the MPDs (Δ*G*_*e*_ > 0) and, thus, causes a reduction in the nucleation rate of the MPDs and an increase in the average diameter of the minority phase droplets/particles, as shown in Figs 5 and 6. The experimental results for the average particle diameter are also shown in Fig. 6. The acceptable agreement between the numerical results and the experimental ones indicates that the model describes the microstructure evolution process well.

In summary, the influence of the ECPs on the solidification of immiscible alloys was investigated. The results demonstrate that the ECPs show a negligible effect on the temperature profile, the convective flow of the melt and the spatial motions of the MPDs. The ECPs affect the microstructure formation mainly through changing the energy barrier for the nucleation of the MPDs. When the matrix liquid has a lower electric conductivity compared to the MPD, the ECPs lead to a decrease in the energy barrier for the nucleation of the MPDs and promote the formation of a sample with finely dispersed microstructure. When the matrix liquid has a higher electric conductivity compared to the MPD, the ECPs cause an increase in the energy barrier for the nucleation and are against the obtaining of a sample with well dispersed microstructure.

## Methods

**Figure 7:**
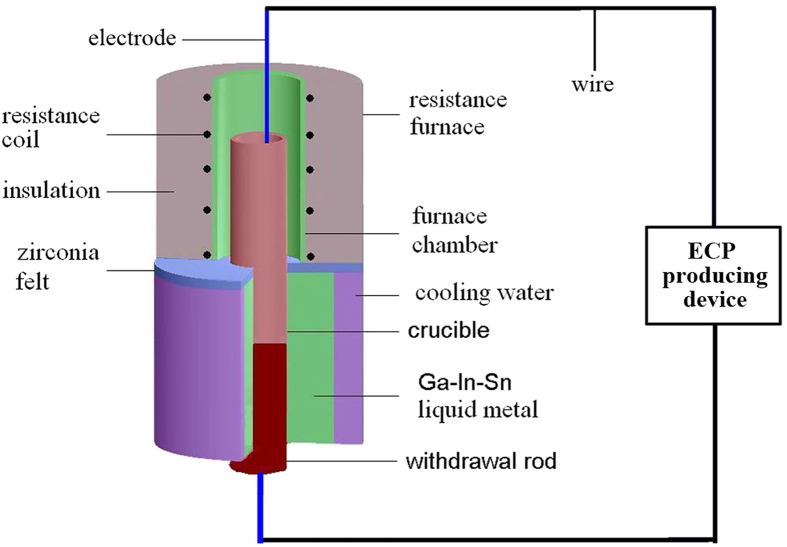
Schematic diagram showing the solidification process under the effect of the ECPs.

Pure Cu (99.99 wt.%), Bi (99.9 wt.%) and Sn (99.9 wt.%) were used as the raw materials. They were melted in a vertical Bridgman type furnace with the ECPs producing device (see Fig. 7). Continuous solidification experiments were performed with Cu-Bi-Sn alloys. The experimental procedures are as follows: Firstly, pure Cu, Bi and Sn were melted in an electric resistance furnace and heated to 1173 K. The melt was held at this temperature for 40 minutes to form a single phase liquid. Then, the ECPs were applied to the melt and the crucible was withdrawn into a bath of the Ga-In-Sn liquid alloy at the rate of 10 mm/s. The samples have a cylindrical shape. The diameter and the length of the samples are 6 and 120 mm, respectively. The samples were cut longitudinally along the central axis to prepare the metallographic specimens. The microstructure characterizations were made by using a scanning electron microscope (Hitachi S-3400N) in the back-scattered mode. SISC IAS V8.0 software developed by the Chinese Academy of Sciences was used for the quantitative metallographic analysis to determine the size distribution and the average diameter of the minority phase particles.

## Additional Information

**How to cite this article**: Hongxiang, J. *et al*. Influence of electric current pulses on the solidification of Cu-Bi-Sn immiscible alloys. *Sci. Rep*. **5**, 12680; doi: 10.1038/srep12680 (2015).
